# Efficacy and safety of compassionate use for rare diseases: a scoping review from 1991 to 2022

**DOI:** 10.1186/s13023-023-02978-x

**Published:** 2023-11-28

**Authors:** Jiayu Wu, Yang Yang, Jiaxin Yu, Luyao Qiao, Wei Zuo, Bo Zhang

**Affiliations:** 1 Department of Pharmacy and State Key Laboratory of Complex Severe and Rare Disease, Peking Union Medical College Hospital (Dongdan Campus), Chinese Academy of Medical Sciences and Peking Union Medical College, No.1 Shuaifuyuan Wangfujing Dongcheng District, Beijing, 100730 China; 2 State Key Laboratory of Bioactive Substance and Function of Natural Medicines, Institute of Material Medica, Chinese Academy of Medical Sciences and Peking Union Medical College, Beijing, 100050 China

**Keywords:** Compassionate use, Rare disease, Orphan drug, Case reports, Expanded access

## Abstract

**Background:**

Compassionate use is a system that provides patients with expedited access to drugs which has not yet been approved, but currently in clinical trials. The investigational drugs have been authorized for compassionate use in cases involving patients suffered from life-threatening diseases and with no alternative treatments. For instance, patients afflicted with highly heterogeneous rare diseases are eligible for treatment assistance through the compassionate use program. This study aims to investigate the characteristics of compassionate use in the context of rare diseases, evaluate the efficacy and safety of compassionate use for rare diseases, and analyze the marketing approval of investigational drugs.

**Methods:**

The case reports/case series of compassionate use were collected by conducting searches on Embase, PubMed, Web of Science, CNKI and SinoMed, spanning from January 1991 to December 2022. Subsequently, two independent reviewers evaluated these reports. Case reports/case series that met the inclusion criteria and exclusion criteria were enrolled. Information extracted from these reports and series included patients' basic information, the investigational drug's name, its indication, adverse events, treatment outcomes, and other relevant data.

**Results:**

A total of forty-six studies were included, encompassing 2079 patients with an average age of 38.1 years. Thirty-nine different drugs were involved in 46 studies. Furthermore, neoplasms emerged as the most common therapeutic area for compassionate use in rare disease management (23/46, 50.0%). Regarding the treatment efficacy, four studies reported successful disease resolution, while 35 studies observed symptom improvement among patients. Conversely, four studies documented no significant effects on patients' diseases. Moreover, one study reported worsened results following compassionate use, while the efficacy was not described in 2 studies. Adverse events were reported in 31 studies (67.4%) because of the compassionate use, while no adverse events occurred in 13 studies (28.3%). In other 2 studies, there was no description about whether treatment-emergent adverse events (TEAEs) were happened. 136 patients (6.5%) had Grade 5 adverse events (death), of which 19 deaths (0.9%) were considered to be related to compassionate use. Furthermore, the investigational drugs in 33 studies (33/46, 71.7%) received new drug approval at the end of January 31, 2023.The time lag from the start of the compassionate use to the formal approval of the investigational drug was 790.5 (IQR 359–2199.3) days. We found that in 11 studies, encompassing 9 different drugs, some compassionate use indications had not received regulatory authorities at the end of January 31, 2023.

**Conclusion:**

The current status of compassionate use for rare diseases was clarified systematically in this study. Compassionate use of investigational drug is a significant treatment option for rare disease. In general, compassionate use appears to demonstrate favorable efficacy in the context of rare diseases, with a significant proportion of compassionate use drugs subsequently receiving marketing approval. However, the safety of drugs for compassionate use cannot be fully evaluated due to the safety data were not covered in some enrolled studies. Therefore, the establishment of an adverse event reporting system specific to compassionate use is warranted.

**Supplementary Information:**

The online version contains supplementary material available at 10.1186/s13023-023-02978-x.

## Background

Rare diseases (RDs) have recently garnered increased attention and have become a prominent public health concern. RD refers to a disease that affects a small number of people in a population [1]. The Food and Drug Administration (FDA) defined RDs as conditions affecting < 200,000 individuals in the United States [2], whereas the European Union Regulation on orphan medicinal products defined RDs as conditions affecting < 50 per 100,000 individuals in the European population [3]. With over 7000 rare diseases identified and a continual increase in their recognition, the societal burden of these diseases is mainly reflected in their high mortality rate, disability rate, years of life of lost [4]. In addition, characterized by small and highly heterogeneous patient populations, there are limited or no approved treatments for many rare diseases, so patients may not have access to clinical trials [5]. In such cases, compassionate use (CU) provides a potential way with patients to access unapproved investigational drugs before formal product approval.

CU is a system that provides patients with special access to drugs which has not yet been approved, but currently tested in clinical trials. The prerequisite for CU is that the patients suffer from life-threatening diseases, and are refractory to conventional therapies [6]. The initiation of CU traces back to the Food and Drug Management Modernization Act of 1997 by FDA, [7], which led to the development of a standardized application process for compassionate drug use. Expanded Access Program Report described the specific responsibilities of patients, doctors, drug research and development enterprises, ethics review committees, and the FDA in the application process [7, 8]. The European Medicines Agency (EMA) and the Committee for Medicinal Products for Human Use (CHMP) announced the official guidelines on CU in 2007 [9]. However, by definition of CU, investigational new drugs (INDs) are still in the clinical trial phases and their effectiveness and safety are not entirely clear. The FDA defines three situations for compassionate drug use: Individual patient (including for emergency use) IND, intermediate-size IND (tens to hundreds of people), and treatment IND (after the successful completion of clinical trial and before FDA approval) in a larger population [10]. Currently, there is a paucity of research focusing on the efficacy and safety use of drugs under CU permission [11, 12]. Typically, reports on CU consist of case studies. Patients receiving CU are usually severely ill patients with a variety of conditions. In addition, CU lacks rigorous enrollment criteria to control variables akin to randomized controlled double-blind clinical trials, thus the resultscompassionate administration are often more variable. According to the Expanded Access (Compassionate Use) Submission Data from 2017 to 2021 on the FDA website, the submission of individual patient (including for emergency use) requests for IND account for 99.0% of the total CU submissions [13], further confirming the difficulty of conducting large-scale retrospective analyses on CU. Recognizing the ambiguous status of CU in the treatment of rare diseases, this study analyzed case reports of rare patients who received CU from January 1991 to December 2022. Furthermore, we discuss the relationship between the efficacy and safety of drugs used in CU for RDs. Our aim is to provide valuable insights for clinical practitioners when contemplating the utilization of CU as a potential treatment option for RDs, ultimately striving to enhance the overall outcomes of RD patients.

## Materials and methods

### Search strategy

In this study, we conducted a systematic search on Embase, PubMed, and Web of Science without imposing language restrictions from January 1991 to December 2022. The search terms employed to identify related studies included: (compassionate drug use case) OR (compassionate use case) OR (expand access case report) OR (expand access case) OR (compassionate use case report). In addition, to ensure comprehensive coverage in the preliminary search, we also searched relevant studies on CNKI and SinoMed.

### Inclusion and exclusion criteria

Initially, we identified the case reports with indications of rare diseases that conform to the definition of compassionate use of the FDA and the European Medicines Agency (EMA). For the definition of rare diseases, we referenced the Orphanet database (https://www.orpha.net/consor/cgi-bin/index.php). In the preliminary screening phase, we excluded studies on the CU of therapeutics (unapproved surgical treatment methods, etc.) and medical devices. Thereafter, we meticulously reviewed the short-listed studies and excluded those in the form of meeting abstracts, studies lacking full texts availability, studies that were in any language other than English or Chinese, and multi-drug combinations. Review articles, meta-analyses, and studies that did not report on drug efficacy or safety were also excluded.

### Data extraction

We gathered the following data from case reports or case series: age, gender, patient numbers, country, name and classification of rare disease, drug name, clinical outcome and treatment-emergent adverse events (TEAEs), and the commencement date of CU. The data extraction method for each of the four indexes is shown below.

1. *Classification of rare diseases* diseases are classified according to International Classification of Diseases 11th Revision (ICD-11) [14].
2. *Patient age* fill in the average or median age.
3. *Clinical outcome* according to the reporting of the article, we divided the outcomes into cure, symptoms improved, no significant effect or worsened.
4. *CU start time* CU start time reported in enrolled studies. When there is no description in the text, the publication data were used.

Drug’s generic name, brand name, the date of marketing approved in country or region conducting CU, and indications for drug approved were also collected from official website of authorization of medicines of each country [15–29].

The procedures for data extraction were conducted by two independent reviewers. Data information were collected until January 31, 2023. A pre-established data extraction protocol was adopted. Divergencies that occurred during the data extraction process were resolved by consulting with two reviewers (B Zhang or W Zuo).

### Analysis

We used descriptive statistics to characterize the drugs, diseases, outcomes, and TEAE. The median and interquartile range (IQR) were used to present the descriptive analysis of numerical data. All analyses were conducted using the analytical tools of IBM SPSS Statistics 20.

## Results

### Selection of studies

**Fig. 1:**
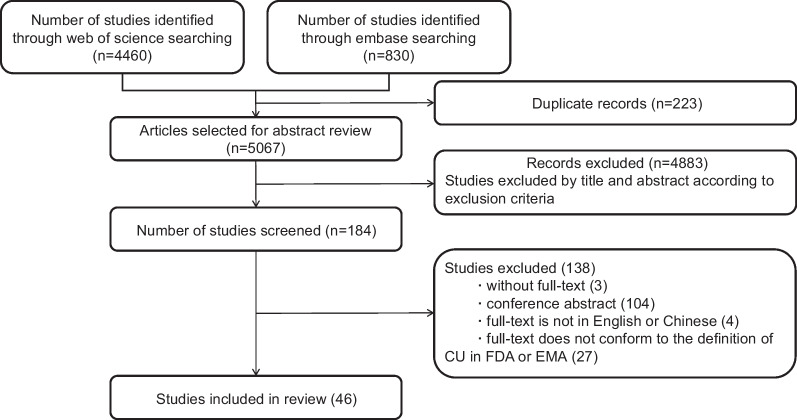
Flow chart of inclusion of studies

6047 titles and abstracts were screened and 980 duplicate studies were excluded. Firstly, based on the inclusion and exclusion criteria, 4883 articles were excluded, resulting in a total of 184 articles being included by title and abstract. Then, upon a comprehensive review of the full text of the initially identified 184 studies, 138 studies were further excluded the unavailability of full-text content (3 articles), conference abstracts (104 articles), publication in languages other than English or Chinese (4 articles), and studies did not meet the definitions of CU of FDA and EMA (27 articles). Finally, forty-six studies were included in this scoping review (see Fig. 1).

### Characteristics of case reports of CU for RDs

This study included a total of 2079 patients with RDs, of which 611 (29.4%) were female and 1468 (70.6%) were male. The average age was 38.1 (0.9–76) years (Additional file 1: Table S1). In addition, 39 different drugs were included in 46 studies. Table 1 shows the characteristics of CU case reports/case series. Neoplasms, accounted for 50.0% (23/46) of the studies, were the most common therapeutic area for target RDs covered by CU studies. CU were also performed in pediatric and geriatric patients; accounting for 13.0% (6/46) and 21.7% (10/46), respectively. In terms of treatment efficacy, Patients with rare diseases were cured in 4 studies, and patients in 35 studies showed symptoms improved. A detailed list of CU cases reports conducted during the survey period is provided in Additional file 1: Table S1.

**Table 1:** Characteristics of CU case reports/case series

	Items	Number of studies	%
Disease classification	01 Certain infectious or parasitic diseases	5	10.9
	02 Neoplasms	23	50.0
	03 Diseases of the blood or blood-forming organs	3	6.5
	04 Diseases of the immune system	2	4.3
	05 Endocrine, nutritional or metabolic diseases	4	8.7
	08 Diseases of the nervous system	3	6.5
	13 Diseases of the digestive system	2	4.3
	15 Diseases of the musculoskeletal system or connective tissue	1	2.2
	19 Certain conditions originating in the perinatal period	1	2.2
	20 Developmental anomalies	2	4.3
Age, years	0–8 years	6	13.0
	> 8–18 years	8	17.4
	> 18–60 years	22	47.8
	> 60 years	10	21.7
Treatment effect	Cured	4	8.7
	Symptoms improved	35	76.1
	No significant effect	4	8.7
	Worsened	1	2.2
	No description	2	4.3
TEAE	Yes	31	67.4
	None	13	28.3
	No description	2	4.3

*TEAE* treatment-emergent adverse events

### Treatment effect and basis of CU cases reports/case series

Table 2 lists the treatment effects and basis of CU case reports, as well as the details of CU. After reading through all the eligible studies, five reasons for CU were summarized: (1) The clinical trial support, or the patient applied for a CU project following the completion of a clinical trial that had good treatment effect; (2) Case report or small series of research literature support; (3) Pharmacological action (such as target) support; (4) Preclinical study support; (5) Similar indications support (The drug has good efficacy and safety for other similar indications). A single CU often has more than one basis for support (Table 2). Clinical trials support was the leading basis of CU accounting for 67.4% (31/46), followed by pharmacological action support at 52.2% (24/46).

**Table 2:** Treatment effect and basis of CU case reports

No	Drug Name	Disease	Basis of compassionate drug use^a^	Treatment effect	CU detail
1	Defibrotide [30]	Hepatic veno-occlusive disease	1 + 3	Cure	The patient was an infant under 1 year old and does not meet the clinical trial conditions
2	Cinacalcet [31]	Oncogenic osteomalacia	3	No significant effect	The patient presented with hypocalcemia due to Cinacalcet’s pharmacological action
3	IV^b^ Ribavirin [32]	Severe adenovirus disease	2	No significant effect	Intravenous injection is still not approved in the FDA. No AEs^c^ during treatment
4	ONC201[33]	Pediatric diffuse intrinsic pontine glioma	1	Symptoms improved	No AEs during treatment
5	Nivolumab [34]	Pediatric glioblastoma	6	Worsened	The patient has Grade 4–5 AE (Brain edema death), and there were reports of AE in other studies
6	Miransertib [35]	PIK3CA-related overgrowth syndrome	1 + 2 + 3	Symptoms improved	No serious AEs
7	Nirogacestat [36]	Desmoid tumors	1	Symptoms improved	No serious AEs
8	Sunitinib [37]	Metastatic alveolar soft part sarcoma	2	No significant effect	Sunitinib has serious skin toxicity, which has been reported in other literatures. The patient presented with necrosis of the skin transplant requiring necrectomy and skin grafting
9	Nedosiran [38]	Primary hyperoxaluria 1	1	Symptoms improved	No serious adverse reactions
10	Haemonine [39]	Haemophilia B	3	Symptoms improved	No serious adverse reactions
11	AL101 [40]	Desmoid tumors	1	Symptoms improved	Grade 3 diarrhea occurred in both patients; however, this adverse event was short-lived and resolved with dose modification
12	Favipiravir [41]	Ebola virus disease	1	No significant effect	The effect of treatment was not statistically significant, except for its influence on survival time
13	Ruxolitinib [42]	Chronic eosinophilic leukemia	2	Symptoms improved	No AEs during treatment
14	Bosentan [43]	Buerger disease or thromboangiitis obliterans	1	Cure	No AEs during treatment
15	Lorlatinib [44]	Inflammatory myofibroblastic tumour	3	Symptoms improved	This case demonstrates a role of lorlatinib in the treatment of TPM4-ALK-rearranged IMT despite failure of entrectinib
16	Defibrotide [45]	Hepatic Veno-occlusive disease	2 + 3	Symptoms improved	Most patients thus had evidence of persistent or intermittent grade 1 or 2 bleeding. Grade 3–4 adverse events during therapy consisted of sepsis; pulmonary edema; CMV^d^ infection; hypotension; etc., but a causative temporal relationship to any grade 3 or 4 toxicity with defibrotide could not be shown
17	Bedaquiline [46]	Multidrug-resistant tuberculosis	1	Symptoms improved	19 SAEs were reported in 14 patients
18	Gemtuzumab Ozogamicin (GO)^e^ [47]	Acute myeloid leukemia, high-risk myelodysplastic syndrome, or acute promyelocytic leukemia	1 + 2	No description	GO was voluntarily withdrawn from the US market in 2010 after a phase III study evaluating GO in combination with chemotherapy in previously untreated adult AML failed to show clinical benefit versus chemotherapy alone
19	Imatinib mesylate [48]	Gastrointestinal stromal tumors	1	Symptoms improved	After the end of clinical trial of imatinib, the patient continued to apply for medication through CU program
20	Human recombinant antithrombin (rhAT)^f^ [49]	Hereditary antithrombin deficiency	2 + 3	Symptoms improved	No serious AEs
21	Ropeginterferon alpha-2b (Besremi) [50]	Classical Ph (−)^g^ myeloproliferative neoplasms	1 + 3	Symptoms improved	Most therapy-related side effects were mild, and there was no treatment discontinuation attributable to intolerable adverse events
22	Aflibercept [51]	Colorectal cancer	1 + 3	Symptoms improved	No serious AEs
23	Hydralazine/Valproate (TRANSKRIP™) [52]	Myelodysplastic syndrome	1 + 3	Symptoms improved	No serious AEs
24	Polatuzumab Vedotin [53]	Diffuse large B-cell lymphoma	1	Symptoms improved	No serious AEs
25	Letermovir [54]	Cytomegalovirus infection	1	Symptoms improved	Six deaths were reported; The local researchers did not classify it as related to Letemovir, only one case occurred during Letemovir because of grade 4 GVHD^h^
26	Pemetrexed [55]	Peritoneal mesothelioma	1	Symptoms improved	The most reported SAEs^i^ for the total expanded access program were dehydration (7.2%), nausea (5.2%), and vomiting (4.9%)
27	Recombinant human interleukin 7 (rhIL-7)^j^ [56]	Multifocal leukoencephalopathy	2 + 3	Symptoms improved	No AEs during treatment
28	Recombinant hirudin [57]	Heparin-induced thrombocytopenia	3	Symptoms improved	No AEs during treatment
29	Imatinib [58]	Chordoma	1 + 3	Symptoms improved	Grade 3–4 AE occurred in 7 of 48 patients
30	Fasudil [59]	Amyotrophic lateral sclerosis	3 + 4 + 5	Symptoms improved	No AEs during treatment
31	Inotuzumab Ozogamicin [60]	B acute lymphoblastic leukemia	1 + 3	Symptoms improved	No AEs during treatment
32	Lorlatinib [61]	Papillary thyroid cancer	3	Symptoms improved	No AEs during treatment
33	Gemtuzumab Ozogamicin [62]	CD33 + acute myeloid leukemia	1 + 3	Symptoms improved	All 24 treated patients developed grade 3–4 neutropenia and/or thrombocytopenia
34	Abiraterone Acetate [63]	Prostate cancer	1	Symptoms improved	Most common grade 3 to 4 AE were anemia (13.9%), hypokalemia (7.3%), fatigue (6.8%), and pain (6.3%). Median duration of treatment was 5.3 months (interquartile range: 2.8–10.3). The main study limitation is its retrospective design
35	Imatinib [64]	Advanced gastrointestinal stromal tumour	1	Symptoms improved	No serious AEs
36	Avapritinib [65]	Gastrointestinal stromal tumour	1	Symptoms improved	No serious AEs
37	Ulixertinib [66]	BRAF D594G cutaneous melanoma	1 + 3	Symptoms improved	The patient accessed ulixertinib via an expanded access program (NCT04566393) as she was ineligible for active clinical trials at the time. No serious AEs
38	Difluoromethylornithine [67]	Bachmann-Bupp syndrome	1 + 3 + 4	Symptoms improved	No AEs during treatment
39	Alpelisib [68]	CLOVES syndrome	2 + 3 + 4	Symptoms improved	No AEs during treatment
40	Risdiplam [69]	Spinal muscular atrophy (SMA)^k^	1 + 2	No description	This article mainly discusses safety of risdiplam. Gastrointestinal disorders were the most common AEs. For patients with SMA1, 30 AEs were reported in 13 cases with 2 serious AEs in 1 patient. For SMA2, 100 AEs were documented in 31 case reports, including 8 serious AEs in 2 patients
41	Avapritinib [70]	Mucosal melanoma and thymic carcinoma	1 + 3 + 5	Symptoms improved	Case 1: grade 2 vasculitis and grade 2 uveitis occurred, in the absence of predisposing factors. Case 2: grade 2 haematologic toxicity occurred, including anaemia and thrombocytopaenia
42	Lumasiran [71]	Primary Hyperoxaluria Type 1	1	Cure	No serious AEs
43	Alpelisib [72]	Extensive venous malformations	1 + 3 + 4	Cure	No serious AEs
44	Avacopan [73]	Antineutrophil Cytoplasmic Antibody–Associated Vasculitis	1	Symptoms improved	No AEs during treatment
45	Bulevirtide [74]	HDV-related cirrhosis	1 + 3	Symptoms improved	No AEs during treatment
46	Asfotase Alfa [75]	Childhood Hypophosphatasia	3	Symptoms improved	No side effects were observed except for mild injection site reactions (erythema, pain, lipohypotrophy, and lipohypertrophy)

^a^(1) The clinical trial support, or the patient participated in the clinical trial with good treatment effect and applied for CU program after the trial; (2) Case report or small series of research literature support; (3) Pharmacological action (such as target) support; (4) Preclinical study support; (5) Similar indications support.

^b^IV: Intravenous injection

^c^AEs: adverse events

^d^CMV: cytomegalovirus

^e^GO: Gemtuzumab Ozogamicin

^f^rhAT: Human recombinant antithrombin

^g^Ph (−): Classical Philadelphia chromosome-negative

^h^GVHD: graft-versus-host disease

^i^SAEs: serious adverse events

^j^rhIL-7: Recombinant human interleukin 7

^k^SMA: Spinal muscular atrophy

### TEAEs and serious adverse events (SAEs) during CU

Patients in 31 studies (67.4%) of CU were found to have definite TEAEs, and patients in 13 studies (28.3%) had no TEAEs. Two studies were not categorized because there was no description of whether TEAEs were happened or not. The detailed TEAEs were displayed in Additional file 1: Table S1, because some studies did not classify the adverse events according to the Common Terminology Criteria for Adverse Events 5.0 (CTCAE 5.0), or the case descriptions were not comprehensive and specific. Table 3 shows the number of TEAE cases and patients reported by the case reporters of CU according to CTCAE classification. We reviewed the 136 (6.5%) patients that experienced Grade 5 adverse events (death), and found that of these, 19 deaths were considered to be related to the investigational drug treatment judged by the authors of case reports or case series enrolled (Table 3).

**Table 3:** Summary of CTCAE case reports and number of the drug-related deaths

Drug Name	Disease	Number of patients^a^	AE grade	TEAE	Drug-related deaths
IV^b^ Ribavirin [32]	Severe Adenovirus Disease	5	5	Severe Adenovirus Disease (n = 3, 60.0%)	–
Nivolumab [34]	Pediatric glioblastoma	1	5	Malignant cerebral edema and herniation (n = 1, 100%)	1
Miransertib [35]	Pik3ca-related overgrowth syndrome	2	1	Hyperlipidaemia (n = 1, 50.0%)	–
				Loose stools (n = 1, 50.0%)	
Nirogacestat [36]	Desmoid tumors	1	2	Diarrhea (n = 1, 100.0%)	–
AL101 [40]	Desmoid tumors	1	1	Fatigue (n = 1, 100.0%)	–
			2	Nausea (n = 1, 100.0%)	–
				Weight decreased (n = 1,100.0%)	
			3	Diarrhea (n = 1,100.0%)	–
Lorlatinib [44]	Inflammatory myofibroblastic tumour	1	1	Oral stomatitis, insomnia, paronychia (n = 1,100.0%) each	
			2	Hyperlipidaemia (n = 1,100.0%)	
Defibrotide [45]	Hepatic veno-occlusive disease	19	1–2 3–4	Nausea (n = 6, 31.6%)	–
				Transient mild systolic hypotension (n = 5, 26.3%)	
				Fever (n = 5,26.3%)	
				Abdominal cramping (n = 3, 15.8%)	
				Vasomotor symptoms (n = 1, 5.3%)	
				Sepsis (n = 7, 36.8%)	–
				Pulmonary edema (n = 5, 26.3%)	
				Cytomegalovirus infection (n = 2, 10.5%)	
				Hypotension (n = 2, 10.5%)	
				Respiratory failure (n = 2, 10.5%)	
				Diarrhea, bronchospasm, alveolar hemorrhage, renal failure, gastrointestinal bleeding, supraventricular arrhythmia, and encephalopathy (n = 1,5.3%) each	
Bedaquiline [46]	Multidrug-resistant tuberculosis	82	5	Respiratory failure (n = 3, 3.6%) Cardiopulmonary failure (n = 2, 2.4%) Peripheral oedema, Nephrotic syndrome, Tuberculosis, Committed suicide, Tuberculosis disease progression, and Myocardial infarction (n = 1, 1.2%) each	2
Gemtuzumab Ozogamicin [47]	AML^c^, high-risk myelodysplastic syndrome, or acute promyelocytic leukemia	331	3–4 5	Anemia (n = 142, 42.9%) WBC^d^ count decrease (n = 120, 36.3%) Platelet count decrease (n = 118, 35.6%) Febrile neutropenia (n = 120, 36.3%) Disease progression (n = 48, 14.5%) Lymphocyte count decrease (n = 81, 24.5%) Hypokalemia (n = 66, 19.9%) Neutrophil count decrease (n = 79, 23.9%) Lung infection (n = 13, 3.9%)	–
				Disease progression (n = 46, 13.9%) AML (n = 16, 4.8%) Sepsis (n = 7, 2.1%) Respiratory failure (n = 5,1.5%) Intracranial hemorrhage (n = 1, 0.3%) Pulmonary hemorrhage, respiratory distress, sepsis, and multiple organ dysfunction syndrome (n = 2, 0.6%) each	15
Ropeginterferon Alpha-2b (Besremi) [50]	[Ph (−)]^e^ myeloproliferative neoplasms	9	1	Anemia, thrombocytopenia, depression, and bone pain (n = 1, 11.1%) each Mucositis (n = 2, 22.2%) Dizziness (n = 2, 22.2%) Alopecia (n = 3, 33.3%)	–
			2	Anemia, leukopenia, and neuropathy (n = 2, 22.2%) each Headache, insomnia, and transaminitis (n = 1, 11.1%) each	–
			3	Anemia, leukopenia, thrombocytopenia, and transaminitis (n = 1, 11.1%) each	–
			4	Anemia (n = 1, 11.1%)	–
Aflibercept [51]	Colorectal cancer	5	1	Alopecia, hypertension, dysphonia, epistaxis conjunctival toxicity, and acute neurotoxicity (n = 1, 20.0%) each Diarrhoea (n = 2, 40.0%)	–
			2	Proteinuria, palmar plantar erythrodysesthesia, alopecia, fatigue, nausea, diarrhoea, neutropenia,respiratory infection, and mucositis (n = 1, 20.0%) each Hypertension (n = 2, 40.0%) Hyporexia (n = 2, 40.0%)	– –
			3	Neutropenia, hypertension, and fatigue (n = 1, 20.0%) each	
Hydralazine and Valproate (TRANSKRIP™) [52]	Myelodysplastic syndrome	14	1–2	Distal tremor (n = 9, 64.3%) Edema (n = 8, 57.1%) Nausea (n = 7, 50.0%) Drowsiness (n = 6, 42.9%) Headache (n = 5, 35.7%) Skin rash (n = 1, 7.1%)	–
Letermovir [59]	CMV^f^ infection	80	5	Graft-versus-host disease (n = 5, 6.2%) CMV infection (n = 2, 2.5%) Adenovirus colitis, influenza infection, leukemia relapse, cardiac arrest, and adenovirus pneumonia (n = 1, 1.2%) each	1
Gemtuzumab Ozogamicin [62]	AML	24	3–4	Neutropenia and/or thrombocytopenia (n = 24, 100%)	–
			5	Veno-occlusive disease (n = 1, 4.2%) Septic shock (n = 1, 4.2%)	–
Abiraterone Acetate [63]	Prostate cancer	368	3–4	Pain (n = 23, 6.3%) Nausea (n = 5, 1.4%) Vomiting (n = 2, 0.5%) Fatigue (n = 25, 6.8%) Peripheral edema (n = 3, 0.8%) Cardiac disorders (n = 3, 0.8%) Anemia (n = 51, 13.9%) Neutropenia (n = 7, 1.9%) Thrombocytopenia (n = 15, 4.1%) Liver enzymes (n = 13, 3.5%) Hypokalemia (n = 27, 7.3%) Hypertension (n = 13, 3.5%)	–
Avapritinib [65]	Gastrointestinal stromal tumour	1	2	Bilateral lower limb oedema (n = 1, 100.0%)	–
Imatinib [58]	Chordoma	48	≤ 2	Oedema (n = 22, 45.8%) Gastritis (n = 1, 2.1%)	–
			≥ 3	Oedema (n = 2, 4.2%) Nausea (n = 4, 8.4%) Neutropenia (n = 1, 2.1%)	
Ulixertinib [66]	BRAF D594G cutaneous melanoma	1	1 2	Mucositis, rash, and diarrhea (n = 1, 100.0%) each	–
				Intermittent visual floaters (n = 1, 100.0%)	–
Avapritinib [70]	Mucosal melanoma and thymic carcinoma	4	2	Vasculitis, uveitis, anaemia, and thrombocytopaenia (n = 1, 25.0%) each	–

^a^Number of patients: Refers to the total number of people using drugs in CU

^b^IV: Intravenous injection

^c^AML: Acute myeloid leukemia

^d^WBC: white blood cell

^e^Ph (−): Classical Philadelphia chromosome-negative

^f^CMV: Cytomegalovirus

### Rate of marketing approval for drugs used in CU, and the interval from CU to approval

**Fig. 2:**
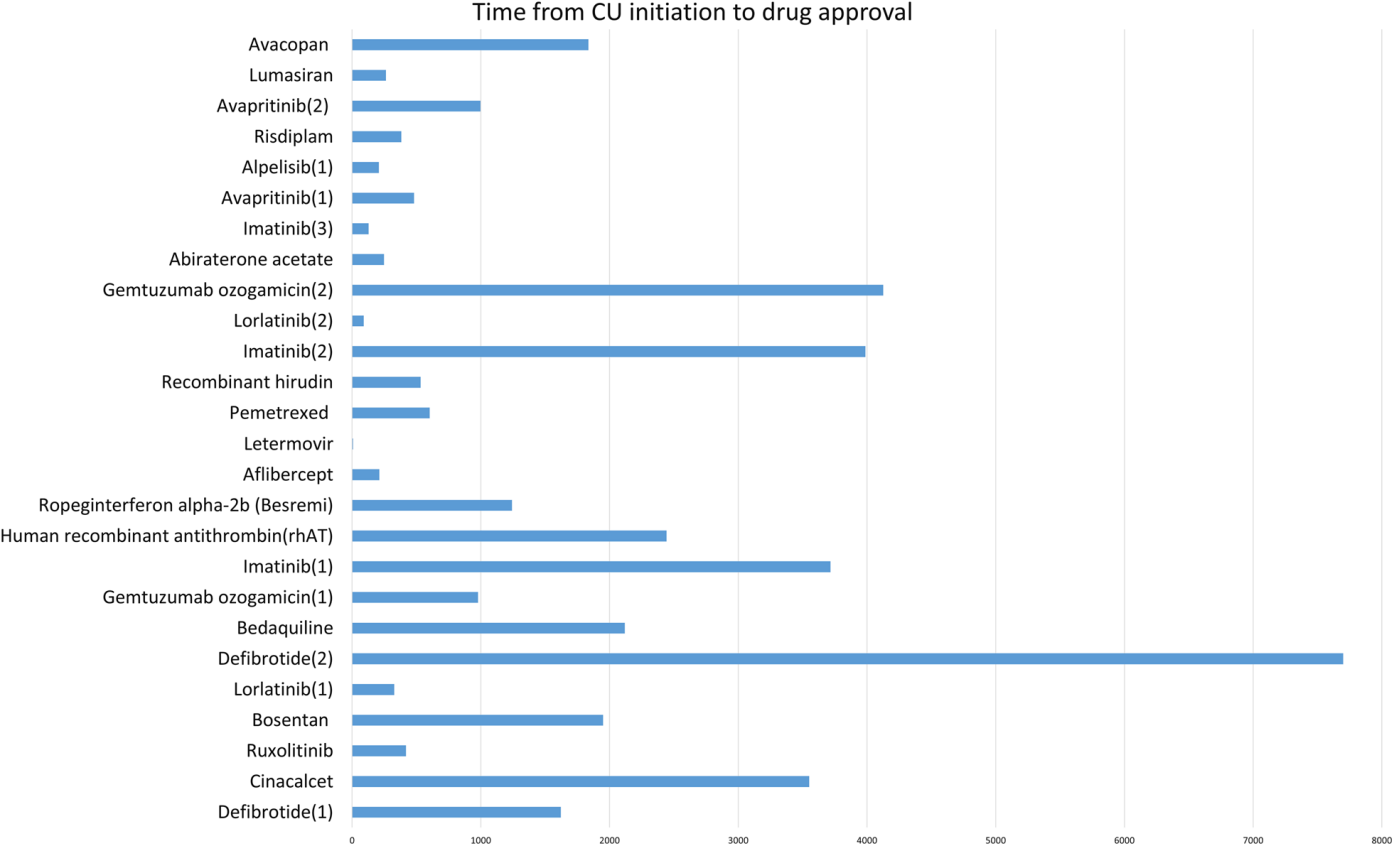
Time from CU initiation to drug approval

By the end of January 31, 2023, we counted the countries where the drugs were used in CU and when they were approved for marketing in that country, and found that 33 of the 46 drugs used in CU (71.7%) had been granted for marketing approval (Additional file 1: Table S1). Simultaneously, we studied the relevance between CU initiation and date of getting approved in 33 drugs. The median number of days from the start time of CU to the formal approval of the investigational drugs were 790.5 (IQR 359–2199.3) days (Fig. 2). Since the exact starting time of several CU case reports cannot be obtained, we adopted the publication time of the studies. Seven drugs were approved earlier than the publication time of the studies, so the 7 studies were not included in the time comparison (Additional file 1: Table S1). Additionally, we conducted an evaluation to determine whether the indications of approval were consistent with those of CU drugs (Additional file 1: Table S1). Our analysis revealed that 11 of the drugs reported in the studies were on the market for indications other than for which the drug had been used as CU.

## Discussion

Although several reports on the result of CU program for individual drugs have been published [76–78], to the best of our knowledge, this study marks the first to specifically concentrate on Cu for RDs. The challenge of engaging patients in the initiation of clinical trials is pronounced due to the rarity of these diseases and the limited number of affected individuals, particularly when considering the inclusion of infants and the elderly [5, 79]. In this study, the efficacy, basis and safety of compassionate use of investigational drugs for rare diseases were reviewed in 46 studies.

First, in terms of efficacy (Table 1), the CU in most of the enrolled studies (39/46, 84.8%) had been showed good efficacy. The relationship between the basis of drug use and the efficacy were further explored. There are drugs whose efficacy have been proved by clinical trials. Good clinical outcomes were seen in 27 out of 30 CU studies, which drugs were used for CU for reason 1(the clinical trial support). When a patient has exhausted all available treatment options and necessitates CU, the physician may employ a structured approach to select a suitable drug for compassionate use, such as referring to RCT research results, pharmacological effects, etc.

In terms of marketing approval, there have been some previous reports [80, 81], focusing on the marketing of a drug in specific countries. McKee et al*.* [80] conducted an investigation with a specific focus on single patient INDs, including individual patient INDs for emergency use, and thus excluded intermediate-size and treatment INDs. They reported an approval rate of 33% within 5 years following the initial IND submission. The research by Maeda et al. explored the market situation of CU drugs in Japan [81]. In this study, we focused on the marketing of CU drugs for rare diseases in the world. Among the 46 CUs conducted, 33 (71.3%) drugs had been approved at the end of January 2023. The process from the commencement of CU to the attainment of marketing approval spanned an average of 790.5 days, (IQR 359–2199.3). The indications of the 11 drugs reported in the studies on the market are different from those of the drug used as a CU. Although CU is gradually gaining public acceptance, it is still difficult for RD patients to obtain access to unauthorized medicine [82]. Therefore, there is an urgent need to abbreviate the time interval from CU to market approval to expedite patients' access to these drugs. We believe that a more efficient global CU system should be in place to offer patients faster and more flexible access to innovative and novel investigational drugs. At the same time, a comprehensive, open-access database is imperative to record the AEs and treatment-effects during CU, which could facilitate follow-up research. When administering drugs for compassionate use to patients, it is important to take a variety of factors into account, such as drug mechanism and clinical trial results, consultation of relevant literature, the assurance of efficacy, safety, and the avoidance of severe adverse effects.

This study is subject to certain limitations and has notable strengths. Firstly, this is a retrospective survey of published information, comparing the efficacy and safety profile of different drugs in the case report/case series. And the information of some studies was incomplete, particularly with regard to the safety data, which could pose challenges in drawing a definite conclusion. Secondly, CU studies may have publication bias, and cases with poor efficacy and safety might not have been published. But anyway, as the first retrospective study specifically focused on CU for RDs, this research establishes a foundational reference for clinical decision-making, and contributes real-world data. Since there hasn't been much review studies on CU for RDs, this study fills in some of the gaps and provides valuable insights into the effectiveness and safety of CU for RDs.

## Conclusion

As the first retrospective survey of CU of unlicensed drugs for RDs, this study has clarified the current status and character of CU, and discussed its efficacy and safety. The treatment of rare diseases is an important reason for some compassionate use, and involves many kinds of rare diseases. In general, CU has demonstrated effectiveness, although it cannot directly prove the safety of drugs used for CU, it can still reflect existing safety issues. A noteworthy finding is that 17 (36.9%) drugs in CU received marketing approval within 5 years of the CU being in place. It provides some reference value for developing future drug policies and enhances our understanding of the prevailing scenario. In short, the efficient treatment of rare diseases remains a lot of challenge, and some regions have not established compassionate use programs. Ultimately, considering the profound impact CU of unlicensed drugs can can have on patients with RDs, further research on safety and efficacy in this is to be encouraged, thereby granting early access to much needed treatments during the lengthy marketing approval process.

### Supplementary Information

**Additional file 1**. **Table S1**: List and details of CU studies. *Adopted the publication time of the studies, so these studies were not included in the time comparison.

## Data Availability

The datasets supporting the conclusions of this article are included within the article and additional file.

## Abbreviations

CU: Compassionate use
IND: Investigational new drugs
RD: Rare diseases
AE: Adverse events
FDA: Food and Drug Administration
EMA: European Medicines Agency
CHMP: Committee for Medicinal Products for Human Use
TEAE: Treatment-emergent adverse events
ICD-11: Classification of Diseases 11th Revision
RCT: Randomized controlled trial
CTCAE 5.0: Common Terminology Criteria for Adverse Events 5.0
IV: Intravenous injection
CMV: Cytomegalovirus
GO: Gemtuzumab ozogamicin
rhAT: Human recombinant antithrombin
Ph (−): Classical philadelphia chromosome-negative
GVHD: Graft-versus-host disease
SAEs: Serious adverse events
rhIL-7: Recombinant human interleukin 7
SMA: Spinal muscular atrophy
WBC: White blood cell
