# Supplementary material for: Efficacy and safety of compassionate use for rare diseases: a scoping review from 1991 to 2022
Source: Orphanet J Rare Dis. 2023 Nov 28;18:368. doi: 10.1186/s13023-023-02978-x (PMC10685565; doi:10.1186/s13023-023-02978-x)
Supplement: Supplementary file 1 — Additional file 1. Table S1: List and details of CU studies. *Adopted the publication time of the studies, so these studies were not included in the time comparison. [file 13023_2023_2978_MOESM1_ESM.pdf]

Table S1 List and details of CU studies

No.	Study title	Drug name	Average age	Number of males	Number of females	Total number of patients	Indication	Disease classification	Treatment effect	Clinical outcome	TEAES	CTCAE 5.0 classification	Country	Drug approval date	CU initiation date	Time from CU initiation to drug approval	Whether the approved indication is consistent with the CU disease
1	Hepatic Veno-Occlusive Disease during chemotherapy for nephroblastoma: Successful and safe treatment with defibrotide. Report of a clinical case	Defibrotide(1)	0.92	1	/	1	Hepatic Veno-Occlusive Disease during chemotherapy for nephroblastoma	13 Diseases of the digestive system	Cure	From day 4 of treatment with defibrotide laboratory tests gradually improved rising to a complete normali zation in about twenty days. After day 7 of treatment with defibrotide and supportive care, an improvement in the clinical signs and symptoms was observed and therefore defibrotide was discontinued.	/	/	Italy	2013/10/18	2009/5/11	1621	Y
2	Tumor-induced rickets in a child with a central giant cell granuloma: A case report	Cinacalcet	3	1	/	1	Oncogenic osteomalacia	15 Diseases of the musculoskeletal system or connective tissue	No significant effect	Compassionate use of cinacalcet, a calcimimetic drug that suppresses PTH secretion and by reducing PTH induced phosphate excretion may raise serum phosphate, was tried.This treatment was unsuccessful and was discontinued after a month because of hypocalcemia.	This treatment was unsuccessful and was discontinued after a month because of hypocalcemia.	Unable to judge	Spain	2018/10/24	2009/2/1	3552	N
3	Intravenous ribavirin treatment for severe adenovirus disease in immunocompromised children.	IV Ribavirin	4.6	2	3	5	Severe Adenovirus Disease	01 Certain infectious or parasitic diseases	No significant effect	Intravenous ribavirin was not effective for all children with severe adenovirus disease in this series or in the litera ture, therapy is unlikely to be of benefit if begun late in the course of the infection	The remaining 3 children died of adenovirus disease.	5	USA	N/A	1997/7/1	N/A	N/A
4	First clinical experience with DRD2/3 antagonist ONC201 in H3 K27M–mutant pediatric diffuse intrinsic pontine glioma: A case report	ONC201	10	/	1	1	H3 K27M-mutant pediatric diffuse intrinsic pontine glioma	02 Neoplasms	Symptoms improved	These tumors remained stable in size over the subsequent 6 months on MRI. To date, no adverse events have been observed or reported due to ONC201. The patient remains clinically improved as of the latest follow-up visit, 19 months after starting ONC201 and 22 months from diagnosis	/	/	USA	N/A	2019/4/5	N/A	N/A
5	Severe cerebral edema following nivolumab treatment for pediatric glioblastoma: Case report	Nivolumab	10	/	1	1	Pediatric glioblastoma	02 Neoplasms	Worsened	Nivozumab treatment failed, resulting in brain edema, severe immune reaction and eventual death	strong treatment response may result in rapid inflammation and tissue breakdown, leading to malignant cerebral edema and herniation events.	5	USA	2014/12/22	2016/6/1*		N
6	Clinical experience with the AKT1 inhibitor miransertib in two children with PIK3CA-related overgrowth syndrome	Miransertib	10.5	1	1	2	PIK3CA-related overgrowth syndrome	19 Certain conditions originating in the perinatal period	Symptoms improved	A subsequent reduction in seizure frequency was observed along with reported improved cognitive engagement in school and at home. Parental-reported quality of life measures, determined on clinical questioning at patient visits, improved.	Treatment was very well tolerated with no adverse efects apart from loose stools (grade 1 CTCAE v.5.0),	1	Ireland	N/A	2021/2/27	N/A	N/A
7	Safety and efficacy of gamma-secretase inhibitor nirogacestat (PF-03084014) in desmoid tumor: Report of four pediatric/young adult cases	Nirogacestat	10.6	2	2	4	Desmoid tumors	02 Neoplasms	Symptoms improved	After a median of 13.5 months (range 6-18), three had durable benefit: a complete response (Case 1); a partial response (Case 2); stable disease (Case 3). The fourth had disease progression after a partial response.	Grade 2 Diarra appears in case 4	2	USA	N/A	2020/8/11	N/A	N/A
8	Necrosisof a skin autograft after short-term treatment with sunitinib in a 14-year-old girl with metastatic alveolar soft part sarcoma of the thigh	Sunitinib	14	/	1	1	Metastatic alveolar soft part sarcoma	02 Neoplasms	No significant effect	sunitinib (Sutent ®) was started on a compassionate use basis. 2 weeks later the patient presented with necrosis of the skin transplant requiring necrectomy and skin grafting.	It has serious skin toxicity, skin ulcer, and second skin transplantation	Severe or medically significant	Austria	2009/5/29	2010/5/31*		N
9	Nedosiran dramatically reduces serum oxalate in dialysis-dependent primary hyperoxaluria 1: A compassionate use case report	Nedosiran	17	/	1	1	Primary hyperoxaluria 1	05 Endocrine, nutritional or metabolic diseases	Symptoms improved	Monthly Nedosiran injections led to dramati cally decreased plasma oxalate levels, decreased frequency of weekly hemodialysis sessions from 6 to 3, and deferral of combined kidney and liver transplant.	No adverse events were reported except for temporary discomfort at the injection site	mild	USA	N/A	2020/7/1	N/A	N/A
10	Clinical efficacy, safety and pharmacokinetic properties of the plasma-derived factor IX concentrate Haemonine(®) in previously treated patients with severe haemophilia B	Plasma-derived factor IX concentrate Haemonine	26.9	14	/	14	Haemophilia B	03 Diseases of the blood or blood-forming organs	Symptoms improved	The overall response was deemed excellent for all patients (92.9%) except one (good). The investigator assessed overall efficacy as excellent for all patients and tolerability as excellent for 13 (92.9%) patients and as good for one patient.	The intensity of the AEs was mild in four, moderate in three and severe in three cases. At study end, seven AEs had completely recovered.	/	Germany	2009/1/2	2011/8/3*		Y
11	Activity of the gamma secretase inhibitor al101 in desmoid tumors: A case report of 2 adult cases	AL101	27	/	1	1	Desmoid tumors	02 Neoplasms	Symptoms improved	Imaging at cycle 21 day 13, approximately 1.5 years after AL101 initiation, revealed a PR (the longest diameter of tumor decreased from 191 mm to 76 mm, representing a 60% decrease) that has been maintained over the past 2.6 years. The patient has continued treatment and the PR is ongoing. The patient reported improved quality of life, maintained throughout the duration of the treatment, with minimal side effects and short infusion time	Grade 3 diarrhea had a sudden onset, occurring within 1-2 weeks of treatment initiation; it was short-lived and resolved with dose modification	3	Australia	N/A	2021/7/23	N/A	N/A
12	Laboratory Findings, Compassionate Use of Favipiravir, and Outcome in Patients with Ebola Virus Disease, Guinea, 2015 - A Retrospective Observational Study	Favipiravir	30	441	372	813	Ebola Virus Disease	01 Certain infectious or parasitic diseases	No significant effect	In multivariate regression analysis, a higher Ct and a younger age were associated with survival ($P < .001$), while favipiravir treatment showed no statistically significant effect ($P = .11$). However, Kaplan-Meier analysis indicated a longer survival time in the favipiravir-treated group ($P = .015$).	No description	/	Guinea	N/A	2015/2	N/A	N/A
13	Efficacy of ruxolitinib in chronic eosinophilic leukemia associated with t(8;9)(p22;p24) and PCM1-JAK2 fusion gene	Ruxolitinib	31	/	1	1	Chronic eosinophilic leukemia not otherwise specified	02 Neoplasms	Symptoms improved	The patient obtained a complete clinical remission with regression of anemia, leukocytosis, eosinophilia, splenomegaly, andmarrow fibrosis;improvement of thrombocytope nia; and restoration of polyclonal hematopoiesis.	/	/	Italy	2012/8/23	2011/7/1	419	N
14	Effectiveness of bosentan in the treatment of ischemic lesions in a case of thromboangiitis obliterans (Buerger disease): A case report	Bosentan	35	/	1	1	Buerger disease or thromboangiitis obliterans	04 Diseases of the immune system	Cure	Six months after starting bosentan therapy, the pain and trophic lesions in the patient's toes had completely disappeared. Bosentan was well tolerated, without any observed adverse reaction	/	/	Spain	2016/11/15	2011/7/15	1950	N
15	Lorlatinib for the treatment of inflammatory myofibroblastic tumour with TPM4-ALK fusion following failure of entrectinib	Lorlatinib(1)	40	1	/	1	Inflammatory myofibroblastic tumour	02 Neoplasms	Symptoms improved	Compassionate use of the third generation ALK inhibitor lorlatinib resulted in excellent partial response on all disease sites after 2 months, followed by a further 6 months of disease stabilisation.	Lorlatinib was otherwise well tolerated with only mild toxicities of grade 1 oral stomatitis, insomnia, paronychia and grade 2 hyperlipidaemia.	1,2	England	2021/7/26	2020/9/2	327	N
16	Treatment of severe veno-occlusive disease with defibrotide: Compassionate use results in response without significant toxicity in a high-risk population	Defibrotide(2)	40	6	13	19	Hepatic veno-occlusive disease (VOD) after stem cell transplantation (SCT)	13 Diseases of the digestive system	Symptoms improved	Resolution of VOD (bilirubin F2 mg/dL with improvement in other symp toms and signs) was seen in 8 patients (42%). Six of 8 responders survived past day 1 100, contrasted with the 2% predicted survival reported in comparable patients.	Most patients have evidence of continuous or intermittent grade 1 or grade 2 bleeding during treatment. Level 3-4 adverse events during treatment include sepsis; Pulmonary edema; Cytomegalovirus (CMV) infection; hypotension; Respiratory failure; Single observation of diarrhea, bronchospasm, alveolar hemorrhage, renal failure, gastrointestinal bleeding (GI) caused by open rectal ulcer, supraventricular arrhythmia and encephalopathy	1,2,3,4	USA	2016/3/30	1995/3/1	7700	Y
17	Is 6 months of bedaquiline enough? Results from the compassionate use of bedaquiline in Armenia and Georgia	Bedaquiline	40.5	68	14	82	Multidrug-resistant tuberculosis	01 Certain infectious or parasitic diseases	Symptoms improved	Using BDQ through compassion shows relatively good success rate and safety. The high reversal rate may indicate that in some cases, 24-week BDQ is necessary.	19 SAEs were reported in 14 patients.,Of the 10 fatalities, six (60%) were reported as being related to advanced TB. A possible causal relation ship to BDQ was reported in two cases, and to other anti-tuberculosis drugs in six cases.	severse	Armenia and Georgia	2019/1/18	2013/4/1	2118	Y
18	Safety of gemtuzumab ozogamicin as monotherapy or combination therapy in an expanded-access protocol for patients with relapsed or refractory acute myeloid leukemia	Gemtuzumab ozogamicin(1)	42.2	331	/	331	Acute myeloid leukemia, high-risk myelodysplastic syndrome, or acute promyelocytic leukemia	02 Neoplasms	No description	/	68%, 39% and 33% of patients had corresponding treatment interruption. 52%, 22% and 22% of the patients in the monotherapy group, the combined therapy group and the APL group respectively had a level 5 AE with full cause-and-effect relationship. Corresponding level 3 and 4 treatment-related adverse events were reported in 60%, 55% and 78% of patients. Hepatotoxicity was found in 5 cases: venous occlusive disease (n=4) and drug-induced liver injury (n=1). GO is generally well tolerated in patients with R /RAML or APL. The most common treatment-related AE ≥ grade 3 is hematology AE	3,4,5	USA	2017/9/1	2014/12/27	979	Y
19	Cerebral relapse of metastatic gastrointestinal stromal tumor during treatment with imatinib mesylate: Case report	Imatinib(1)	47	1	0	1	Gastrointestinal stromal tumors	02 Neoplasms	Symptoms improved	This case illustrates that the brain can be a sanctuary site to treatment of GISTs with imatinib. Maintaining dosing of imatinib in the face of isolated sites of disease progression is also important, as other metastatic sites may still be sensitive.	No description	/	Australia	2013/4/18	2003/2/14	3716	Y
20	Use of recombinant human antithrombin in patients with congenital antithrombin deficiency undergoing surgical procedures	Human recombinant antithrombin(rhAT)	49.6	3	2	5	Hereditary antithrombin deficiency	03 Diseases of the blood or blood-forming organs	Symptoms improved	There was no clinical evidence of thrombosis or bleeding. Four of the five patients had postopera tive duplex ultrasound studies of the lower extremities, which showed no evidence of acute thrombosis. Four patients were tested for antirecombinant rhAT antibod ies postoperatively and were negative	Except for the spontaneous elimination of skin pigmentation at the site of drug infusion, there were no drug-related adverse events	mild	USA	2009/5/6	2002/8/28	2443	Y
21	Real-world experience with Ropeginterferon-alpha 2b (Besremi) in Philadelphia-negative myeloproliferative neoplasms	Ropeginterferon alpha-2b (Besremi)	51	5	4	9	Classical Philadelphia chromosome-negative [Ph(-)] myeloproliferative neoplasms	02 Neoplasms	Symptoms improved	·Both the molecular response and complete blood count remission rates were 62.5%.<br>·symptom amelioration and spleen size reduction.<br>·significantly attenuated plasma levels of inflammation markers were observed in one particular patient who happened to have normalized spleen size and most remarkable reduction in JAK2 mutant allele burden, indicating all-around improvement in every aspect of this case.<br>·plasma hepcidin levels increased in two-thirds of PV patients, illustrating the potential of Ropeg to restore normal regulation of erythropoiesis.	1.Hematological: Leukopenia;Anemia;Thrombocytopenia ;<br>2.Non-hematological: Depression;lopecia;Bone pain;Neuropathy;Headache;Insomnia;Mucositis;Dizziness;Transaminitis	1,2,3,4	Taiwan, China	2020/5/27	2017/1/1	1242	Y
22	Efficacy and safety of aflibercept in metastatic colorectal cancer pretreated with bevacizumab: A report of five cases	Aflibercept	55	2	3	5	Colorectal cancer	02 Neoplasms	Symptoms improved	The present study contrasts the efficacy and safety results obtained from the pivotal VELOUR trial, and confirms that aflibercept, used in routine clinical practice outside of the clinical trial environment, is active and well-tolerated following bevacizumab treatment	The patient showed grade 2 hypertension, hypotension, nausea and diarrhea, and grade 3 fatigue,	1,2,3	Spain	2013/9/1	2013/2	212	Y
23	Encouraging results with the compassionate use of hydralazine/valproate (TRANSKRIP™) as epigenetic treatment for myelodysplastic syndrome (MDS)	hydralazine/valproate(TRANSKRIP™)	55.2	8	6	14	Myelodysplastic syndrome	02 Neoplasms	Symptoms improved	five patients remained on the treatment, one with partial response and four with complete response. The median OS was not reached in the eight patients who saw a clinical benefit from the treatment, in comparison to an OS of 7 months in the six patients who had no treatment. The combination of hydralazine and valproate is safe and effective in MDS, and its further testing is highly desirable.	All 14 patients were evaluated for toxicity. Most of the toxicity is transient, grade 1-2, and no dose reduction or treatment interruption is required. The most common toxicity was distal tremor (n=9, 64.2%), followed by edema (n=8, 57%), nausea (n=7, 50%), lethargy (n=6, 42.8%) and headache (n=5, 35%); Only one patient developed a rash and disappeared without treatment	1,2	Mexico	N/A	2009/11/1	N/A	N/A
24	Compassionate use of polatuzumab vedotin in patients with relapsed/refractory diffuse large B-cell lymphoma: Report of one case and review of literature	Polatuzumab vedotin	57	1	/	1	Diffuse large B-cell lymphoma	02 Neoplasms	Symptoms improved	the patient had received 6 cycles of pola combined with rituximab since December 2019. Unexpected adverse events were not found during the treatment. The evaluation of clinical efficacy was complete remission after the end of treatment. The progression -free survival time was more than 13 months with follow - up until January 2021.	The first infusion reaction symptoms of chills and fever occurred during the infusion of rituximab in the first treatment course	mild	China	N/A	2019/12/1	N/A	N/A
25	Letermovir for Secondary Prophylaxis of Cytomegalovirus Infection and Disease after Allogeneic Hematopoietic Cell Transplantation: Results from the French Compassionate Program	Letermovir	57	42	38	80	Cytomegalovirus infection	01 Certain infectious or parasitic diseases	Symptoms improved	letermovir given as a SP may prevent a new CMV reactivation in a high-risk patient population and can be administered for several weeks, providing a bridge between the pre-emptive or therapeutic treatment of a CMV episode and CMV specific immune reconstitution, giving time for tapering immunosuppressants. Prospective studies are required to confirm these results	The first infusion reaction symptoms of chills and fever occurred during the infusion of rituximab in the first treatment course	2,4	France	2018/1/8	2018/1/1	7	Y
26	Open-label study of pemetrexed alone or in combination with cisplatin for the treatment of patients with peritoneal mesothelioma: Outcomes of an expanded access program	Pemetrexed	58	60	34	94	Peritoneal mesothelioma	02 Neoplasms	Symptoms improved	Pemetrexed with or without cisplatin had a favorable safety profile, and the disease control rate (CR + PR + SD) of 71.2% in the subset of patients with peritoneal mesothelioma indicated activity in this patient population.	Neutropenia and febrile neutropenia were each reported in < 1% of patients in the EAP. The most commonly reported nonhematologic SAEs in the EAP, irrespective of causality, includ ed dehydration (7.2%), nausea (5.2%), vomiting (4.9%), dysp nea (3.8%), and pulmonary embolism (2.4%).	Severe or medically significant	USA	2004/2/4	2002/6/12	602	Y
27	Treatment of Progressive Multifocal Leukoencephalopathy With Interleukin 7	Recombinant human interleukin 7 (rhIL-7)	61	1	/	1	Progressive multifocal leukoencephalopathy	08 Diseases of the nervous system	Symptoms improved	More than 2 years after initial presentation, she remains stable and serum JCV PCR is undetectable. This case demonstrates successful treatment of PML in a patient with idiopathic CD4 lymphocytopenia and highlights the potential benefits of IL-7 and MVC in the treatment of PML	/	/	Norway	N/A	2018/4/7	N/A	N/A
28	USE OF RECOMBINANT HIRUDIN AS ANTITHROMBOTIC TREATMENT IN PATIENTS WITH HEPARIN-INDUCED THROMBOCYTOPENIA	Recombinant hirudin	62.8	2	4	6	Heparin-induced thrombocytopenia	03 Diseases of the blood or blood-forming organs	Symptoms improved	After discontinuation of heparin and the onset of recombinant hirudin, clinical evolution was uneventful in all patients, with no recurrence of thromboembolism, limb amputation, or hemorrhagic complication. The aPIT ratio varied from 1.8 to 3.5 (median 2.4) throughout administration of recombinant hirudin. Platelet count rose from nadir (median value 60 x log, 15 to 90) to above 100 x 109/L in every patient within 3-6 days (median 5), after discontinuation of heparin.	/	/	France	1997/3/13	1995/9/27	533	Y
29	Imatinib in advanced chordoma: A retrospective case series analysis	Imatinib(2)	64	31	17	48	Chordoma	02 Neoplasms	Symptoms improved	the activity of imatinib in locally advanced and metastatic chordoma, and tumor growth arrest was more than 70% in advanced patients. The median duration of the reaction lasted for nearly 10 months, and more than 20% of the patients did not progress after 18 months	42 patients (87.5%) reported any level of toxicity. Edema is the most common adverse event, which occurs at any level in 24 /48 (50%) patients, but G3 only occurs in two cases. 21 /24 (87.5%) patients with edema added a small amount of diuretics, and 12 /24 (50%) patients had to reduce the dose of imatinib to 400 mg/day. 14 /48 patients (29%) had transient renal function damage, and all patients were relieved after temporary interruption of treatment and water supplement. The toxicity that led to the final stop of treatment included: gastrointestinal intolerance in four patients (G2-3 nausea and vomiting in three patients, G3 gastritis in another patient).	2,3	Italy	2013/7/1	2002/8/1	3987	N
30	Compassionate Use of the ROCK Inhibitor Fasudil in Three Patients With Amyotrophic Lateral Sclerosis	Fasudil	65.3	1	2	3	Amyotrophic lateral sclerosis	08 Diseases of the nervous system	Symptoms improved	Blood pressure, heart rate and routine laboratory tests were constantly controlled. All three subjects tolerated the Fasudil infusions well without any obvious side effects. Interestingly, the slow vital capacity showed a significant increase in one of the patients	/	/	Germany	N/A	2017/5/1	N/A	N/A
31	Inotuzumab ozogamicin is effective in relapsed/refractory extramedullary B acute lymphoblastic leukemia 11 Medical and Health [illegible]	Inotuzumab ozogamicin	66.5	1	1	2	B acute lymphoblastic leukemia	02 Neoplasms	Symptoms improved	These two cases suggest a possible key role of IO in the setting of advanced CD22 positive ALL, and underline its potential activity also in patients with EM involvement, relapsed after or refractory to conventional chemotherapy	/	/	Italy	2017/6/29	2018/3/30*		Y
32	Targeting EML4-ALK gene fusion variant 3 in thyroid cancer	Lorlatinib(2)	67	1	/	1	ALKPapillary thyroid cancer	02 Neoplasms	Symptoms improved	Within two weeks, the level of thyroglobulin decreased by about 75%. After 11 weeks of treatment with loratinib, CT scan showed partial remission (PR), and the total number of target lesions decreased by 37%. Similarly, after 7 months of starting loratinib treatment, the continuous PR was determined, and the total number of target lesions decreased by 38% compared with the previous CT scan (61% [illegible]	/	/	Netherlands	2019/5/6	2019/2/4	91	N
33	Fractionated gemtuzumab ozogamicin and standard dose cytarabine produced prolonged second remissions in patients over the age of 55 years with acute myeloid leukemia in late first relapse	Gemtuzumab ozogamicin(2)	68	11	13	24	CD33+ acute myeloid leukemia	02 Neoplasms	Symptoms improved	·The response rate was 75%, including complete remission (CR) in 16 patients and CR with incomplete platelet recovery (CRp) in two patients.<br>·Two-year overall survival was 51% (95% CI: 28-69) and 2 years relapse-free survival was 51% (95%CI: 25-72).<br>·Duration of second CR was longer than first CR in 9 out of 18 patients.<br>·Minimal residual disease was negative in evaluable patients in second CR, particularly in NPM1 mutated cases.	neutropenia; thrombocytopenia;infections	3,4	France	2018/4/19	2007/1/1	4126	Y
34	Abiraterone acetate post-docetaxel for metastatic castration-resistant prostate cancer in the Belgian compassionate use program	Abiraterone acetate	73	368	0	368	Prostate cancer	02 Neoplasms	Symptoms improved	The median time of PSA and imaging progress was 4.1 months and 5.8 months respectively. The median overall survival period is 15.1 months	Mostcommon grade 3 to 4 adverse events were anemia(13.9%),hypokalemia(7.3%),fatigue(6.8%),and pain(6.3%).Median duration of AA treatment was 5.3months	3,4	Belgium	2011/9/5	2011/1/1	247	Y
35	Ten years of treatment with 400 mg imatinib per day in a case of advanced gastrointestinal stromal tumor	Imatinib(3)	74	1	/	1	Advanced gastrointestinal stromal tumour	02 Neoplasms	Symptoms improved	In the continuous follow-up (once every 2 months in the first 6 months, and once every 6 months thereafter), the regression of tumor was observed and has continued to the present. The patient's physical performance has always been good.	Side effects include periorbital edema, sudden muscle spasm of toes and fingers, bone and joint pain, tremor, pale skin color and mild anemia.	Moderate	Germany	2001/11/7	2001/7/1	129	Y
36	Preseptal cellulitis, intraocular inflammatory reaction and corneal persistent epithelial defect as side effects of avapritinib	Avapritinib(1)	76	/	1	1	Gastrointestinal stromal tumour	02 Neoplasms	Symptoms improved	The patient remained stable after adjusting the dose of avapitinib (100mg, once a day) for more than 1 year.	Two months after receiving avapitinib treatment, the right eye developed pre-uterine cellulitis, which subsequently developed into intraocular inflammatory reaction and continuous corneal epithelial defect, edema of bilateral lower limb G2, and bilateral lower limb cellulitis on December 27,	2,3	Spain	2020/9/24	2019/6/1	481	Y
37	Case report: response to the ERK1/2 inhibitor ulixertinib in BRAF D594G cutaneous melanoma	Ulixertinib	43	/	1	1	BRAF D594G cutaneous	02 Neoplasms	Symptoms improved	Ulixertinib provided a clinically meaningful benefit by rendering her disease resectable, and she currently remains disease-free at 1 year.	Grade 1 mucositis, grade 1 rash, grade 1 diarrhea, and intermittent grade 2 visual floaters, the ulixertinib dose was adjusted numerous times over the course of several weeks and ultimately held for a period of 2 weeks, after which it was resumed at 150 mg twice daily with no further adverse effects	1,2	USA	N/A	2020/12/1	N/A	N/A
38	Improvement of dermatological symptoms in patients with Bachmann-Bupp syndrome using difluoromethylornithine treatment	Difluoromethylornith	6.3	1	1	2	Bachmann-Bupp syndrome	05 Endocrine, nutritional or metabolic diseases	Symptoms improved	Two patients diagnosed with Bachmann-Bupp syndrome using oral Difluoromethylornithine, we observed an improvement in classic dermatological findings. Both patients showed increased hair growth and cessation of previously recurring follicular cysts.	/	/	USA	N/A	2020/10/1	N/A	N/A
39	Alpelisib to treat CLOVES syndrome, a member of the PIK3CA-related overgrowth syndrome spectrum	Alpelisib(1)	2	1	/	1	CLOVES syndrome	20 Developmental anomalies	Symptoms improved	Alpelisib reduced the volume of the cystic lymphangioma by approximately 31%. Although it had little effect in the gluteal region, it achieved a slight reduction in fatty overgrowth and prevented progression	/	/	Spain	2020/7/27	2020/1/1	208	N
40	Short-term safety results from compassionate use of risdiplam in patients with spinal muscular atrophy in Germany	Risdiplam	24.7	48	63	111	Spinal muscular atrophy(SMA)	08 Diseases of the nervous system	No description	/	3 patients (1 patient with SMA1 and 2 patients with SMA2) experienced at least 1 SAE.The most common non-serious AEs in SMA1 were gastrointestinal disorders: diarrhea was observed 3 times (10.0% of all AEs), abdominal pain and salivary hypersecretion both occurred 2 times (6.7% of all AEs). In SMA2, diarrhea was the most common non-serious AE (9 events, 9.0% of all AEs), followed by headache (6 events, 6.0% of all AEs), and aphthous ulcer, constipation, nausea and circumstance or information capable of leading to medication error (all 4 events, 4.0% of all AEs).	Severe or medically significant	Germany	2021/3/29	2020/3/12	382	Y
41	Differential activity of avapritinib in patients with metastases from mucosal melanoma and thymic carcinoma harbouring KIT exon 17 mutations: Initial experience from a Compassionate Use Program in Italy	Avapritinib(2)	42.3	2	2	4	Mucosal melanoma and thymic carcinoma	02 Neoplasms	Symptoms improved	3/4 patients responded, 1/4 did not respond, along with possible hypotheses to justify such differences and potential implications for precision oncology.	Case 1: grade 2 vasculitis and grade 2 uveitis occurred, in the absence of predisposing factors. Case 2: grade 2 haematologic toxicity occurred, including anaemia and thrombocytopaenia.	2	Italy	2020/9/24	2018/1/1	997	N
42	Case Report: Sustained Efficacy of Lumasiran at 18 Months in Primary Hyperoxaluria Type 1	Lumasiran	13	1	/	1	Primary Hyperoxaluria Ty	05 Endocrine, nutritional or metabolic diseases	Cure	The patient had a rapid and sustained decrease in urinary oxalate/creatinine ratio, with a mean reduction after lumasiran administration of about 70%. During the 18 months long follow-up, urinary oxalate remained low, reaching nearly normal values. Plasma oxalate also decreased dramatically. Normal levels were reached immediately after the first dose and remained consistently low thereafter. During the same follow-up period, eGFR remained stable at about 60 ml/min/1.73 m2, but no new kidney stones were observed. Existing kidney stones did not increase in size.	In terms of safety, no severe or serious adverse event was observed, apart from temporary and asymptomatic episodes of macroscopic hematuria after the first four Lumasiran injections.	Mild or Moderate	Belgium	2020/11/19	2020/3/1	263	Y
43	Repurposing alpelisib, an anti-cancer drug, for the treatment of severe TIE2-mutated venous malformations: Preliminary pharmacokinetics and pharmacodynamic data	Alpelisib(2)	15.3	2	1	3	Extensive venous malforn	20 Developmental anomalies	Cure	Pain was controlled, gait improved, size of the abnormal venous network decreased, and coagulopathy dramatically improved. Drug exposure was highly variable, suggesting that alpelisib dosing should be individualized to patient's characteristics and guided by therapeutic drug monitoring	Adverse events associated to alpelisib treatment included an episode of self-resolved headache with normal cerebral computed tomography scan (patient 3), two episodes of superficial thrombophlebitis, likely related to underlying disease (patients 1 and 3), and spontaneously resolved sole pain (patient 2).	Mild or Moderate	Canada	2020/7/1	2022/7/25*		N
[illegible]	Compassionate Use of Avacopan in Difficult-to- [illegible]	[illegible]	[illegible]	[illegible]	[illegible]	[illegible]	Antineutrophil [illegible]	[illegible]	[illegible]	compassionate-use avacopan allowed to ameliorate disease and to significantly reduce and stop steroid [illegible]	[illegible]	[illegible]	[illegible]	[illegible]	[illegible]	[illegible]	[illegible]
